# Screening for undiagnosed atrial fibrillation using a single-lead electrocardiogram at primary care visits: patient uptake and practitioner perspectives from the VITAL-AF trial

**DOI:** 10.1186/s12875-023-02087-5

**Published:** 2023-06-30

**Authors:** Steven J. Atlas, Jeffrey M. Ashburner, Yuchiao Chang, Leila H. Borowsky, Patrick T. Ellinor, David D. McManus, Steven A. Lubitz, Daniel E. Singer

**Affiliations:** 1 Division of General Internal Medicine, Massachusetts General Hospital, 100 Cambridge St, Suite 1600, Boston, MA 02114 USA; 2 Harvard Medical School, Boston, MA USA; 3 Demoulas Center for Cardiac Arrhythmias and Cardiovascular Research Center, Massachusetts General Hospital, Boston, MA USA; 4 Department of Medicine, University of Massachusetts Medical School, Worcester, MA USA

**Keywords:** Atrial fibrillation, Preventive screening, Primary care, Provider survey

## Abstract

**Background:**

Screening for atrial fibrillation (AF) is appealing because AF is common, when undiagnosed may increase stroke risk, and stroke is preventable with anticoagulants. This study assessed patient and primary care practitioner (PCP) acceptability of screening for AF using a 30-s single-lead electrocardiogram (SL-ECG) during outpatient visits.

**Methods:**

Secondary analyses of a cluster randomized trial. All patients ≥ 65 years old without prevalent AF seen during a 1-year period and their PCPs. Screening using a SL-ECG was performed by medical assistants during check-in at 8 intervention sites among verbally consenting patients. PCPs were notified of “possible AF” results; management was left to their discretion. Control practices continued with usual care. Following the trial, PCPs were surveyed about AF screening. Outcomes included screening uptake and results, and PCP preferences for screening.

**Results:**

Fifteen thousand three hundred ninety three patients were seen in intervention practices (mean age 73.9 years old, 59.7% female). Screening occurred at 78% of 38,502 individual encounters, and 91% of patients completed ≥ 1 screening. The positive predictive value of a “Possible AF” result (4.7% of SL-ECG tracings) at an encounter prior to a new AF diagnosis was 9.5%. Same-day 12-lead ECGs were slightly more frequent among intervention (7.0%) than control (6.2%) encounters (*p* = 0.07). Among the 208 PCPs completing a survey (73.6%; 78.9% intervention, 67.7% control), most favored screening for AF (87.2% vs. 83.6%, respectively), though SL-ECG screening was favored by intervention PCPs (86%) while control PCPs favored pulse palpation (65%). Both groups were less certain if AF screening should be done outside of office visits with patch monitors (47% unsure) or consumer devices (54% unsure).

**Conclusions:**

Though the benefits and harms of screening for AF remain uncertain, most older patients underwent screening and PCPs were able to manage SL-ECG results, supporting the feasibility of routine primary care screening. PCPs exposed to a SL-ECG device preferred it over pulse palpation. PCPs were largely uncertain about AF screening done outside of practice visits.

**Trial registration:**

ClinicalTrials.gov NCT03515057. Registered May 3, 2018.

**Supplementary Information:**

The online version contains supplementary material available at 10.1186/s12875-023-02087-5.

## Background

Atrial fibrillation (AF) is the most common cardiac arrhythmia and is frequently identified and treated by primary care practitioners (PCPs) [1]. The risk of developing AF increases with age, and having AF confers a fivefold increased risk of stroke [1–3], which is largely preventable by long-term use of oral anticoagulants (OACs) [4, 5]. AF is often diagnosed when a patient presents with symptoms that lead to obtaining a confirmatory electrocardiogram (ECG). However, AF may be asymptomatic [6, 7], and patients may first be diagnosed with AF at the time of an acute stroke presentation [8–10]. To forestall such events, there is interest in screening for AF so OAC treatment can be initiated for AF-related stroke prevention [11].

Methods to screen for AF include pulse palpation, standard ECGs, wearable ECG patch monitors, and newer techniques such as wrist-worn wearable technologies and handheld ECGs [12–16]. Screening studies have been performed in various clinical and non-clinical settings [13, 17–20]. Screening using consumer wearable technologies can identify patients with AF, but patients may not follow up with a physician following an abnormal result [14, 15, 21]. An advantage of screening at a primary care visit is that the PCP can immediately initiate evaluation and treatment if AF is identified.

Guidelines differ with regard to screening for AF. The European Society of Cardiology recommend opportunistic screening for AF by pulse palpation or ECG in patients ≥ 65 years of age and consideration of systematic ECG screening in individuals age ≥ 75 years or those at high risk of stroke [11]. Current American Heart Association/American College of Cardiology guidelines do not directly address screening [22]. Though the U.S. Preventive Services Task Force (USPSTF) found the current evidence is insufficient to assess the balance of benefits and harms of screening for AF, it states that pulse palpation is considered to be usual care [23].

To address this uncertainty, the VITAL-AF trial assessed the feasibility and efficacy of population-based screening for AF in older patients using a 30-s handheld single-lead ECG during routine primary care visits. The primary outcomes of the trial have been previously published [24]. In the current paper, we examine patient and PCP acceptability of incorporating a single-lead ECG as part of routine primary care visits, the impact of screening on PCP effort and utilization of 12-lead ECGs, and survey-based perceptions about screening for undiagnosed AF among PCPs.

## Methods

VITAL-AF was a pragmatic cluster-randomized controlled trial with primary care practices as the unit of randomization and individual patients as the unit of analysis. The study took place within the Massachusetts General Hospital Primary Care Practice-Based Research Network, with 16 of 22 practices participating (8 intervention, 8 control). Details of the study methodology and primary outcomes have been previously described [24, 25].

### Patient and PCP eligibility

Patients aged 65 years and older presenting for a visit with a PCP in a participating practice between 7/31/2018 and 10/8/2019 were eligible. Each practice enrolled patients for one year. For pragmatic reasons, all patients meeting age criteria, including those with a history of AF, were included in the trial. However, only patients without a history of AF are included in these analyses. Only encounters with a PCP (physician or advanced practice provider) were included to allow for the rapid management of screening results. For the PCP survey, primary care staff physicians and advanced practice providers from intervention and control practices were included. Resident physicians were not surveyed.

### Intervention protocol

Personnel in intervention practices were trained by research staff about study procedures with monthly refreshers for medical assistants. Eligible patients were mailed a letter approximately two weeks before a scheduled visit informing them about the study and inviting them to participate at their upcoming visit. Patients scheduled in a time frame too short to mail a letter were given a paper information sheet at the visit. When patients checked in, a printed sheet with a unique study identification number and barcode was automatically printed to remind medical assistants that the patient was eligible to participate. Medical assistants then asked eligible patients if they would like to participate in the study while performing routine intake and vital signs assessment. Verbally consenting patients then had vital signs obtained and, in addition, placed their fingers on a single lead AliveCor KardiaMobile ECG device (AliveCor Inc., Mountain View, CA) affixed to an iPad (Apple Inc. Cupertino, CA) to conduct AF screening. The software was configured to read the printed barcode and automatically link the ECG tracing to the participant’s study identification number. All screening was performed by practice medical assistants, not research staff.

Screening results appeared on the device and included the categories: “Possible AF,” “Normal,” “Unclassified,” or “No analysis (unreadable).” Medical assistants were instructed to repeat the single-lead ECG once for “Unclassified” or “No analysis” results and then document the final result in the electronic medical record (EMR) (Epic, Verona, WI) along with other vital signs. A paper form with results was also provided for practices that routinely provided PCPs with written vital sign results. Medical assistants notified PCPs if a patient had a “Possible AF” reading. PCPs were informed that the single-lead ECG was a screening test and did not confer a definitive diagnosis of AF. PCPs managed all subsequent care including whether to obtain a 12-lead ECG during the visit. As part of the study, independent cardiologists reviewed all single-lead ECG tracings within seven days. PCPs were notified if a pre-specified actionable rhythm was identified but not addressed during the visit (Supplemental Table 1).

### Primary care practitioner survey

The survey content was adapted from a prior survey instrument by the study investigators [26]. Survey topics included questions about preferences for screening for AF as part of primary care visits and non-visit based screening. For PCPs from intervention practices, additional questions assessed their experience and perceptions with screening for AF with the SL-ECG including support provided by the study and how well it was integrated into routine practice (Supplemental Material). Following the 1-year enrollment period, eligible PCPs from intervention and control practices were emailed an invitation to a REDcap survey [27]. Up to three reminder emails were sent, and those not responding were mailed a paper survey with a return envelope.

### Outcomes and statistical analysis

Outcomes assessed included AF screening uptake by patients (proportion completing screening at the encounter and patient level), results of single-lead ECGs, utilization of 12-lead ECGs on the same day of a primary care encounter, and survey-based PCP perceptions of AF screening. For patients with more than one screen, the most to least “abnormal” result was ranked “Possible AF”, “Unclassified”, “Normal” and “No Analysis.”

Patients seen during the 1-year enrollment period were assigned to intervention or control groups based on the primary care practice where they were first seen. Analyses of encounters excluded visits by intervention patients to control practices (*n* = 378 encounters) and control patients seen in intervention practices (*n* = 764 encounters). Baseline patient characteristics and 12-lead ECGs obtained on the day of primary care encounters were assessed from EMR data using a centralized data warehouse of inpatient and outpatient health data from the Mass General Brigham network [28]. Patients with prevalent AF before study enrollment were excluded from analyses with the diagnosis based upon the presence of International Classification of Diseases, 10^th^ Revision (ICD-10) codes for AF or atrial flutter using a high specificity electronic algorithm or manually adjudicated by two research nurses who reviewed all relevant EHR data to validate the AF diagnosis [25]. Potential newly diagnosed AF events during the 1-year study period were initially ascertained from EMR data based on an ICD-10 code for AF or atrial flutter or a 12-lead ECG with AF or flutter in the diagnostic statement. A clinical endpoint committee then adjudicated new AF from a manual review of the medical record [24].

Patient and PCP characteristics were compared using chi-square tests, t-tests, and Wilcoxon tests as appropriate. The likelihood of being diagnosed with new AF at a primary care visit was compared between intervention and control practices using a Poisson model with the generalized estimating equation (GEE) approach to account for multiple visits from the same patient. Same-day order of 12-lead ECG tests was compared between intervention and control groups using a Poisson model with GEE to account for both provider and patient clustering. To determine the expected number of same-day 12-lead ECGs in the intervention group, we applied the control group utilization rate to intervention encounters. Clinician survey results were summarized with comparisons to clinicians’ responses in control practices using chi-square tests where applicable. Statistical significance was defined as a 2-tailed *P* value ≤ 0.05, and all analyses were conducted using SAS version 9.4 (SAS Institute, Cary, NC).

## Results

Characteristics of the practices and the 15,393 patients in the screening arm and 15,322 in the control arms are provided in Supplemental Table 1. On average, patients were approximately 74 years old, 60% female and most were non-Hispanic white. Age, gender, race, and comorbid conditions were well-balanced between study arms.

### Uptake of single-lead ECG screening for atrial fibrillation among study patients

Over the 1-year study period, intervention patients without a history of AF had 38,502 encounters in an intervention practice (mean 2.5 visits, median 2 [interquartile range 1–3]) and control patients had 39,686 encounters in a control practice (mean 2.6 visits, median 2 [interquartile range 1–3]). Intervention patients were invited to undergo single-lead ECG screening during 34,138 encounters (88.7%), and screening was performed during 29,952 encounters (77.8%) (Table 1). Patients were more likely to be screened during encounters with staff physicians (Supplemental Table 2).

**Table 1:** Single-Lead (SL) ECG performance and results among intervention patients with practice encounters

	**N**	**Mean (SD) or %**
Intervention Practice Encounters, mean	38,502	2.5 (1.8)
Invited to undergo SL-ECG screening	34,138	88.7%
SL-ECG performed	29,952	77.8%
Patients with clinic visit	15,393	
Any SL-ECG performed	14,047	91.3%
≥ 2 SL-ECG performed	8,061	52.4%
First SL-ECG Screen		
Possible AF	363	2.6%
Unclassified	1,590	11.3%
No Analysis	316	2.2%
Normal	11,778	83.8%
Any SL-ECG Screen^a^		
Possible AF	655	4.7%
Unclassified	2,237	15.9%
No Analysis	187	1.3%
Normal	10,968	78.1%

^a^Uses the most abnormal SL-ECG result during the study period

### Results of single-lead ECG screening and PCP notifications

Overall, 14,047 intervention patients (91.3%) had at least one single-lead ECG performed (8,061 [52.4%] ≥ 2) during the study period. At their first encounter with single-lead ECG screening, 2.6% of patients had an AliveCor algorithm result of Possible AF (11.3% Unclassified, 83.8% Normal, 2.2% No Analysis). Over the study period, 4.7% of patients had at least one Possible AF single-lead ECG result (most abnormal screening result: Unclassified [15.9%], Normal [78.1%], and No Analysis [1.3%]).

Cardiologists reviewed 32,659 single-lead ECG tracings resulting in 165 PCP notifications for potentially significant results. Among these were 131 urgent (Level 1) and 23 less urgent (Level 2) notifications sent a mean of 4.6 days (SD 1.7) after the visit (Supplemental Table 3 lists Level 1 and 2 findings). Among the Level 1 notifications, 129 were due to a finding of AF or atrial flutter without mention in the PCP note or on the EMR problem list. There was one instance of atrioventricular dissociation and one instance of second-degree atrioventricular block, Mobitz II).

### Diagnosis of AF during primary care visits

Though single-lead ECG screening did not significantly increase diagnoses of AF over one year among intervention patients [24], patients in intervention practices were more likely to be diagnosed with new AF at a primary care visit than patients in control practices (0.24% [90 events / 38,009 encounters] versus 0.16% [62 events / 39,246 encounters]; RD 0.08%, 95% CI 0.02–0.14). Among the 90 intervention patients with a new diagnosis of AF at an intervention primary care visit, 60 (66.7%) had an AliveCor automated reading of Possible AF. The positive predictive value of a Possible AF result for new AF among intervention patients was 9.5% (60 out of 635 patients with at least one encounter result of Possible AF prior to AF diagnosis, 95% CI 7.3–12.0%).

### Same day 12-lead ECG utilization

**Fig. 1:**
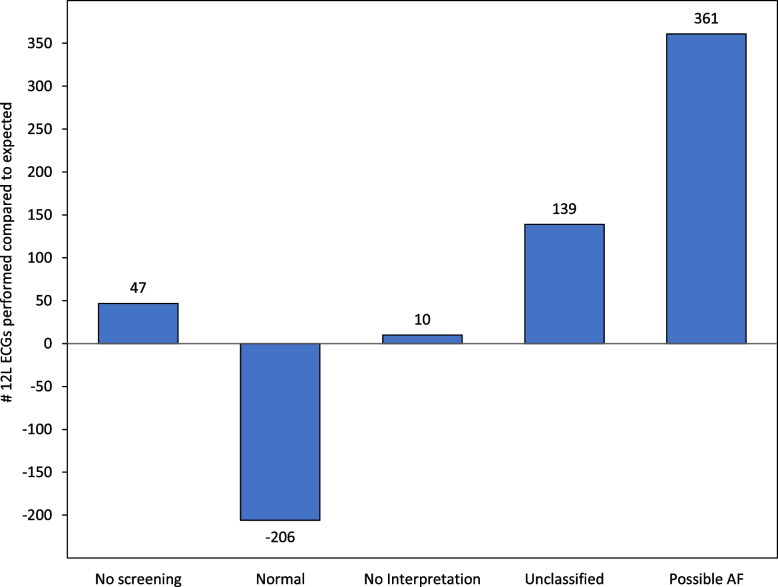
Distribution of excess 12-Lead (12L) ECGs (*n* = 331) performed during intervention arm encounters by single lead ECG screening result. Expected number of same day 12-L ECGs derived by applying the control group utilization rate to intervention encounters

Same day 12-lead ECG utilization occurred slightly more frequently for encounters by intervention patients compared to control patients (7.0% [*n* = 2,702] vs. 6.1% [*n* = 2,424], *p* = 0.07). Among patients in the intervention arm, same-day 12-lead ECGs were ordered more often following encounters with Possible AF (46.6%) than following an Unclassified (9.9%), No Analysis (7.5%), or Normal (5.3%) result. There were 351 more same-day 12-lead ECGs than expected occurring during encounters by intervention patients. Among the excess ECGs, fewer than expected were performed following a normal screening result, whereas more than expected were performed following a non-normal screening result (Fig. 1).

### Primary care clinician survey responses

Among 208 participating PCPs (169 staff physicians, 29 advanced practice providers, and 10 unrecorded), the survey response rate was 73.6% (78.9% intervention; 67.7% control). Intervention PCPs rated the process of integrating single-lead ECG screening into clinical practice favorably (81.4%), even though 57.0% of PCPs estimated that the true positive rate was low (< 30%) (Table 2). Most intervention PCPs (61.6%) reported that single-lead ECG screening led to at least one new diagnosis of AF for one of their patients during the study period, but that came at a perceived increase in ordering of 12-lead ECGs (70.9%) and outpatient rhythm tests (32.9%).

**Table 2:** Survey responses among Intervention PCPs

	Intervention (*N* = 86 PCPs)
	**n (%)**
During the study, did you change how often you obtained 12-lead ECGs? *Yes, I ordered more*	61 (70.9)
During the study, did you change how often you obtained an outpatient rhythm assessment, such as a Holter or patch monitor? *Yes, I obtained more outpatient rhythm assessment tests*	28 (32.9)
How would you rate the overall process of integrating AF screening with the AliveCor Kardia mobile device in your clinical practice for patients 65 and older? *Easy/very easy to integrate*	70 (81.4)
Were you informed about Possible AF screening results for any of your patients? *Yes*	85 (98.8)
How often during the study would you estimate that the AliveCor Kardia mobile screening result led to a new diagnosis of AF for one of your patients? ≥ *1 time*	53 (61.6)
For patients who had a Possible AF screening result at a visit, what percent of the cases would you estimate were true positives? < *30%*	49 (57.0)

**Fig. 2:**
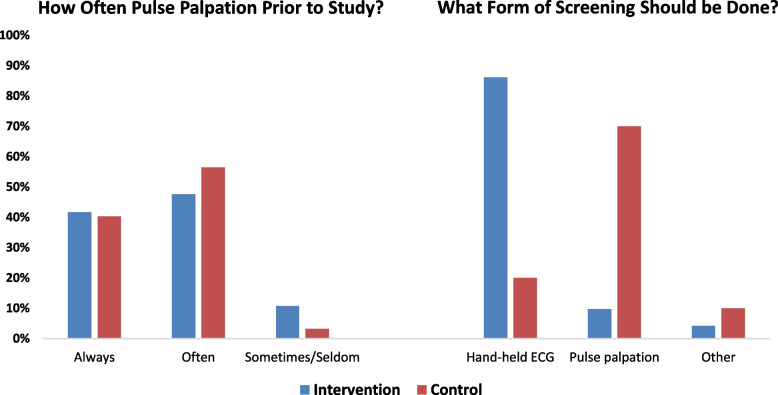
Clinician Survey Responses: A) Prior to the VITAL-AF study, how often did you assess pulse pattern at outpatient primary care visits with asymptomatic patients? (Assessing the pulse pattern includes the clinician palpating the pulse, listening to the heart or performing BP monitoring); B) What form of screening should be done during primary care visits

Comparing responses among intervention and control PCPs, most reported assessing pulse pattern often or always at outpatient primary care visits with asymptomatic patients (89.3% vs. 95.5%, *p*-value 0.16, Fig. 2). Most PCPs felt that AF screening should be done during primary care visits and favored doing it in some patients based on age or risk factors (Table 3). Among those who favored AF screening, single-lead ECG screening was favored by intervention PCPs (86.1%), while control PCPs favored pulse palpation (63.0%) (Fig. 2).

**Table 3:** Primary care practitioner survey responses

	Intervention PCPs	Control PCPs	*P*-value
	**N (%)**	**N (%)**	
Do you think AF screening should be done during primary care visits?	86	62	0.79
Yes, in all adult patients	20 (23.2)	16 (23.9)	
Yes, in some patients based on age or risk factor profile/Other	55 (64.0)	40 (59.7)	
No/Unsure	11 (12.8)	11 (16.4)	
How often do you think AF screening should be done?	72	56	0.14
Once a year during an annual visit	30 (41.7)	28 (50.0)	
At every visit as part of routine VS	39 (54.2)	22 (39.3)	
Do you think that patients at increased risk for AF should be screened outside of office visits for persistent or paroxysmal AF using a one-time 2-week patch monitor?	86	67	0.69
Yes	16 (18.6)	9 (13.4)	
Unsure	39 (45.3)	33 (49.3)	
Do you that that patients at increased risk for AF should be screened outside of office visits for persistent or paroxysmal AF using personal consumer devices?^a^	86	67	0.034
Yes	24 (27.9)	11 (16.4)	
Unsure	48 (55.8)	34 (50.7)	
What is the minimum duration of a single paroxysmal AF episode that would lead you to recommend oral anticoagulation (OAC)?^b^	85	66	0.064
Unsure	41 (48.2)	24 (36.4)	
At least 30 s of AF	31 (36.5)	25 (37.9)	
At least 5 min of AF	5 (5.9)	13 (19.7)	

^a^Personal consumer devices such as the Apple Watch, FitBit, or AliveCor Kardia mobile

^b^For a patient with a CHA2DS2-VASc score of 3 (moderately high stroke risk) and paroxysmal AF

When asked about screening for persistent or paroxysmal AF outside of office visits, both groups were less certain about using patch monitors (47.1% unsure) or consumer electronic devices, (e.g. smart watch, 53.6% unsure). When asked about what duration of a single paroxysmal AF episode would lead them to recommend an oral anticoagulant in a patient at moderately high risk for stroke, about a third said at least 30 s, but more were unsure (Table 3).

## Discussion

We conducted the first trial in the United States to evaluate whether screening for AF at primary care encounters can increase detection in undiagnosed older individuals at least 65 years of age. This randomized controlled trial in over 30,000 individuals from a large primary care practice network demonstrated that screening for AF using handheld single-lead ECGs was feasible as part of routine care when embedded into vital signs assessments. Fully 91% of eligible patients underwent screening during the one-year study period. Single-lead ECG screening modestly increased same day 12-lead ECGs in intervention compared to control patients, driven mainly by more 12-lead ECGs in those with a “possible AF” result. PCPs in both intervention and control practices favored routine screening for AF. Intervention PCPs favored the single-lead ECG over pulse assessment even though they recognized that it generated many false-positive results.

Opportunistic screening for AF with PCPs performing pulse assessment during visits has been shown to increase detection [29]. Screening has also been demonstrated to be feasible in non-clinical settings such as pharmacies and using consumer electronic devices [13, 18, 19, 30, 31]. However, patients identified outside of clinical settings may not follow-up the abnormal result [14, 15, 21]. Our study compared primary care practices that used a single-lead ECG with control practices in which most PCPs performed pulse assessment. Though screening for AF with a single-lead ECG did not increase newly diagnosed cases of AF compared to usual care [24], AF was more likely to be diagnosed in the outpatient setting among patients in the intervention than in the control arm of the trial. Diagnosis of AF in the primary care setting may forestall diagnosis in urgent or emergency care settings.

Patients in our study favorably viewed screening with 91% undergoing at least one single-lead ECG over 1 year. Other studies have also elicited favorable patient views about screening for AF [32, 33]. Similarly, PCPs in the intervention practices favored AF screening with the single-lead ECG over pulse assessment even though most reported that few abnormal single-lead ECG results were true positives. This may have been due to their perceived ease of integrating the single-lead ECG into clinical workflow and the fact that most PCPs reported having had a patient with screen-detected during the study. PCPs in the control practices favored pulse palpation to screen for AF. The physicians’ positive view of single-lead ECG screening may relate to the PCP receiving the automated result and not having to interpret the 30 s tracing itself.

The lack of a significant increase in AF detection among patients in the intervention practices may have been partly due to most PCPs in control practices reporting that they performed pulse assessment at all or most visits. For PCPs who do not routinely perform pulse assessment, single-lead ECGs may still be an efficient way to screen for AF in older patients.

Regarding health care utilization, we found that single-lead ECG AF screening implemented within routine primary care resulted in a modest net increase in 12-lead ECG utilization. The increase in 12-lead ECG utilization observed in those with an abnormal screening result was partially offset by reduced utilization in those with a normal result. Future studies should assess other downstream cardiac testing as well as cardiology and emergency department visits that may be related to single-lead ECG AF screening.

30-s single-lead ECG assessment primarily identifies individuals with persistent AF. Those with paroxysmal AF (PAF) may also be at increased stroke risk [34], and longer monitoring intervals are required to identify screen-detected PAF. When asked, few PCPs in either intervention and control practices favored screening outside of office visits for undiagnosed AF with either a patch monitor or consumer electronic devices. This may reflect uncertainty about the amount of PAF that increases stroke risk [35, 36]. However, almost half of PCPs reported that they would consider anticoagulation for single episodes of at least 30 s or five minutes.

This study has several limitations. We performed the study in a single academic primary care network, and results may not generalize to practices organized in a different way. Our high screening rate reflects workflow adaptation at the practice level and in-person training and refreshers by study staff for medical assistants. Though AF screening was offered at all visits during the study period, the optimal frequency of screening is uncertain. Intervention PCPs knew that single-lead ECGs would be reviewed by cardiologists who would notify them of any concerning findings. Though similar to what is done when obtaining a 12-lead ECG in our network, this level of study oversight may not be feasible as part of routine primary care. Lastly, the 30-s single-lead ECG is most likely to detect persistent AF, where there is little uncertainty regarding the net benefit of anticoagulation for patients with elevated predicted stroke risk. Our intervention study and survey were done before the COVID pandemic, and PCPs favoring office-based over outpatient screening for undiagnosed AF may have changed with increased experience with virtual visits.

## Conclusions

Population-based screening for AF using a single-lead handheld ECG in patients 65 years and older as part of routine outpatient primary care visits is feasible with most patients undergoing screening and was viewed favorably by most PCPs in the intervention practices. Though guideline recommendations differ on the appropriateness of screening for AF, it was supported by PCPs in both intervention and control groups.

## Supplementary Information

**Additional file 1:** **Supplemental Table 1.** Practice and participant characteristics. **Supplemental Table 2.** Single-Lead (SL) ECG performance and results among intervention patients stratified by provider type seen during practice encounters. **Supplemental Table 3.** AliveCor Results Requiring Primary Care Practitioner Notification*.

## Data Availability

The datasets generated during and/or analyzed during the current study are available from the corresponding author [SJA] on reasonable request.

## Abbreviations

AF: Atrial fibrillation
PCP: Primary care practitioner
ECG: Electrocardiogram
SL-ECG: Single-lead electrocardiogram
OAC: Oral anticoagulant
EMR: Electronic medical record
ICD-10: International Classification of Diseases, 10^th^ Revision
GEE: Generalized estimating equation
