# Supplementary material for: Screening for undiagnosed atrial fibrillation using a single-lead electrocardiogram at primary care visits: patient uptake and practitioner perspectives from the VITAL-AF trial
Source: BMC Prim Care. 2023 Jun 30;24:135. doi: 10.1186/s12875-023-02087-5 (PMC10311748; doi:10.1186/s12875-023-02087-5)
Supplement: Supplementary file 1 — Additional file 1: Supplemental Table 1. Practice and participant characteristics. Supplemental Table 2. Single-Lead (SL) ECG performance and results among intervention patients stratified by provider type seen during practice encounters. Supplemental Table 3. AliveCor Results Requiring Primary Care Practitioner Notification*. [file 12875_2023_2087_MOESM1_ESM.docx]

# **Supplemental Table 1. Practice and Participant Characteristics**

|  | **Screening group** | **Control group** |
| --- | --- | --- |
| **Practice characteristics** |  |  |
| Total No. | 8 | 8 |
| Practice location |  |  |
| On campus | 4 | 4 |
| Community practice | 4 | 4 |
| Primary care provider type | 234 | 219 |
| Staff Physician | 108 | 108 |
| Resident Physician/Fellow | 106 | 96 |
| Advanced practice provider | 20 | 15 |
| **Patient characteristics** |  |  |
| Total No. | 15,393 | 15,322 |
| Age, mean (SD), y | 73·9 (6·8) | 74·0 (6·9) |
| Greater than 75 y – No. (%) | 5,758 (37·4) | 5,783 (37·7) |
| Female sex – No. (%) | 9,184 (59·7) | 8·907 (58·1) |
| Race/Ethnicity – No. (%)* |  |  |
| White | 12,688 (82·4) | 12,641 (82·5) |
| Black | 811 (5·3) | 717 (4·7) |
| Hispanic | 334 (2·2) | 304 (2·0) |
| Other | 1,259 (8·2) | 1,347 (8·8) |
| Oral anticoagulant – No. (%) | 518 (3·4) | 530 (3·5) |
| CHA_2_DS_2_VASc score | 3·4 ± 1·4 | 3·4 ± 1·4 |
| ≥ 2 – No. (%) | 14,637 (95·1) | 14,529 (94·8) |
| Current smoker – No. (%) | 723 (4·8) | 784 (5·2) |
| Body mass index, median (IQR), kg/m^2^* | 27·1 (24.0-30.8) | 27.3 (142.-31.0) |
| Antihypertensive – No. (%) | 8,134 (52·8) | 7,843 (51·2) |
| Hypertension – No. (%) | 11,573 (75·2) | 11,519 (75·2) |
| Myocardial infarction – No. (%) | 1,095 (7·1) | 1,075 (7·0) |
| Coronary artery disease – No. (%) | 3,215 (20·9) | 3,141 (20·5) |
| Diabetes mellitus – No. (%) | 3,644 (23·7) | 3,630 (23·7) |
| Heart failure – No. (%) | 1,599 (10·4) | 1,559 (10·2) |
| Prior stroke – No. (%) | 1,278 (8·3) | 1,282 (8·4) |
| Vascular disease – No. (%) | 2,993 (19·4) | 2,969 (19·4) |

* Variables with missing data: race: n=614 (2·0%), BMI: n=115 (0·37%); similar distributions between the two groups.

**Supplemental Table 2. Single-Lead (SL) ECG Performance and Results Among Intervention Patients Stratified by Provider Type seen during Practice Encounters**

|  | **Staff PCP** | **Resident/Fellow** | **Advanced practice provider** |
| --- | --- | --- | --- |
|  | **N (%)** | **N (%)** | **N (%)** |
| Intervention Practice Encounters | 31,109 | 2,502 | 4,891 |
| Invited to undergo SL-ECG screening | 27,990 (90.0) | 2,129 (85.1) | 4,019 (82.2) |
| SL-ECG performed | 24,693 (79.4) | 1,840 (73.5) | 3,419 (69.9) |
| First SL-ECG Screen |  |  |  |
| Possible AF | 304 (2.5) | 29 (3.7) | 30 (3.7) |
| Unclassified | 1,357 (11.3) | 95 (12.2) | 138 (11.1) |
| No Analysis | 256 (2.1) | 29 (3.7) | 31 (2.5) |
| Normal | 10,107 (84.1) | 627 (80.4) | 1,044 (84.0) |
| Any SL-ECG Screen* |  |  |  |
| Possible AF | 548 (4.6) | 44 (5.6) | 63 (5.1) |
| Unclassified | 1,873 (15.6) | 135 (17.3) | 229 (18.4) |
| No Analysis | 156 (1.3) | 16 (2.0) | 15 (1.2) |
| Normal | 9,442 (78.6) | 587 (75.1) | 939 (75.4) |

* Uses the most abnormal SL-ECG result during the study period

**Supplemental Table 3. AliveCor Results Requiring Primary Care Practitioner Notification***

| **AliveCor portal possible entries** | **Qualifying definition** | **Notification level** |
| --- | --- | --- |
| Atrial Fibrillation | Not on problem list | Level 1 |
| Atrial Flutter | Not on problem list | Level 1 |
| V Tach | ≥ 30 seconds | Level 1 |
| Sinus Arrest | Pause ≥ 3 seconds | Level 1 |
| AV Dissociation |  | Level 1 |
| Second Degree AV Block, Mobitz II |  | Level 1 |
| Complete AV Block |  | Level 1 |
| Pacemaker Failure to Capture & Sense |  | Level 1 |
| Pause ≥ 5 seconds in atrial fibrillation* |  | Level 1 |
| Bradycardia with rates averaging < 40 bpm* |  | Level 1 |
| Tachycardia with rates averaging > 150 bpm* |  | Level 1 |
| SVT | ≥ 15 beats | Level 2 |
| Junctional |  | Level 2 |
| Accelerated Junctional Escape Rhythm |  | Level 2 |
| Junctional Tach |  | Level 2 |
| Rapid Ventricular Response |  | Level 2 |
| Non-sustained Ventricular Tach | ≥3 beats, < 30 seconds | Level 2 |
| Short PR Interval, Accelerated AV Conduction | With manifest delta wave | Level 2 |
| Second Degree AV Block, Mobitz I |  | Level 2 |
| Rhythm Consistent with AOO Pacing |  | Level 2 |
| Rhythm Consistent with VOO Pacing |  | Level 2 |
| Rhythm Consistent with DOO Pacing |  | Level 2 |
| Ventricular Preexcitation | Manifest delta wave | Level 2 |
| Short QT Interval | < 350 msec | Level 2 |
| Prolonged QT Interval | > 460 msec | Level 2 |

* Notification protocol: After each AliveCor tracing was reviewed by a study cardiologist, a study nurse would identify any level 1 or level 2 notifications on a daily basis. The study nurse would review the patient’s medical record to identify if the EKG findings was new. Level 1 and 2 notifications were then reviewed by study investigators within 5 business days. If a level 1 notification was felt to be a new diagnosis the PCP was paged with the findings and a templated note was sent to the PCP via the patient’s electronic medical record. For level 2 notifications, a templated noted was sent to the PCP.

**VITAL-AF Study Intervention Practice Survey**

Thank you for participating in the “VITAL-AF study: Screening for Atrial Fibrillation (AF) in an ambulatory clinic population”. This study involved using a single-lead ECG device (AliveCor Kardia mobile) for AF screening when medical assistants checked vital signs in patients 65 years and older.

Now that we have completed the one-year screening period, we are interested in learning about how you check for rhythm abnormalities, including AF, in your patients as well as your experience with this study.

Please note: This survey is voluntary. Proceeding with this survey indicates your consent to participate in the VITAL-AF Study provider survey.

**Part I: Assessing AF in your practice**

1. **Prior to the VITAL-AF study, how often did you assess pulse pattern (palpate pulse, listen to the heart, and/or do your own BP monitoring) at outpatient primary care visits with asymptomatic patients (e.g., no palpitations)?**

1= Never

2= Seldom

3= Sometimes

4= Often

5= Always

1. **Prior to the VITAL-AF study, for how many seconds did you typically assess the pulse pattern?**

0= Not applicable, I don’t assess the pulse pattern

1= 1-10 seconds

2= >10 seconds

1. **During the VITAL-AF study, did you change how you assessed the pulse pattern in asymptomatic patients 65 years and older?**

0= Not applicable, I don’t assess the pulse pattern in asymptomatic patients and didn’t change

1= Yes, I assessed the pulse pattern **less often**

2= No, I didn’t change how I assessed the pulse pattern

3= Yes, I assessed the pulse pattern **more often**

1. **During the VITAL-AF study, did you change how often you obtained 12-lead ECGs?**

1= Yes, I ordered **fewer** ECGs

2= No, I ordered **the same number of** ECGs

3= Yes, I ordered **more** ECGs

1. **During the VITAL-AF study, did you change how often you obtained an outpatient rhythm assessment, such as a Holter or patch monitor?**

1= Yes, I obtained **fewer** outpatient rhythm assessment tests

2= No, I obtained **the same number of** outpatient rhythm assessment tests

3= Yes, I obtained **more** outpatient rhythm assessment tests

**Part II: VITAL-AF Study Support**

1. **I received adequate education and information about the VITAL-AF study, including how to manage screening results.**

1= Strongly disagree

2= Disagree

3= Neutral

4= Agree

5= Strongly agree

1. **During the VITAL-AF study, I felt adequately supported by the VITAL-AF staff, including cardiology back-up, when I had a “Possible AF” or another result for which I wanted assistance.**

0= Not applicable, I never needed assistance or support

1= Strongly disagree

2= Disagree

3= Neutral

4= Agree

5= Strongly agree

**Part III: Integrating the VITAL-AF study into your practice**

1. **How would you rate the overall process of integrating AF screening with the AliveCor Kardia mobile device in your clinical practice for patients 65 and older?**

1= Very difficult/challenging to integrate

2= Somewhat difficult/challenging to integrate

3= Neutral

4= Easy to integrate

5= Very easy to integrate

1. **Were you informed about “Possible AF” screening results for any of your patients?**

0= No

1= Yes

**9.a. If Yes, how were you informed about “Possible AF” screening results? Please select the MOST FREQUENT.**

1= My medical assistant told me about the result

2= I saw the results on a piece of paper from my medical assistant

3= I checked the results in Epic under the rhythm assessment tab

4= I received an InBasket notification from VITAL-AF study staff after the visit

5= I received an email notification from VITAL-AF study staff after the visit

6= Other: ____________________________

1. **Did you receive any Epic In-Basket notifications for positive findings?**

0= No

1= Yes

**10.a. If Yes, did you find the frequency of the Epic In-Basket notifications burdensome?**

0= No

1= Yes

**Part IV: Experience with the Vital-AF Study**

1. **How often during the study would you estimate that the AliveCor Kardia mobile screening result led to a new diagnosis of AF for one of your patients?**

0= Never

1= Once

2= Two times

3= Three times or more

1. **For patients who had a “Possible AF” screening result at a visit, what percent of the cases would you estimate were true positives?**

1= < 30%

2= 30-70%

3= >70%

1. **Did the AliveCor Kardia mobile screening lead you to pay more attention to AF, or heart rhythm assessment in general, in your older patients?**

0= No

1= Yes

8= Unsure

1. **Did the AliveCor Kardia mobile screening lead you to re-evaluate stroke prevention decisions in your patients with known AF?**

0= No

1= Yes

8= Unsure

**Part V: Future Directions for AF Screening**

1. **Do you think AF screening should be done during primary care visits?**

0= No

1= Yes, in some patients based on age or risk factor profile

2= Yes, in all adult patients

3= Other: __________________________________

8= Unsure

**5.a. If yes, what form of screening should be done during primary care visits?**

1= Pulse palpation only (followed by 12-lead ECG if abnormal)

2= Hand-held ECG screening (e.g. AliveCor) device (followed by 12-lead ECG if abnormal)

3= 12-lead ECG screening

4= Other: __________________________________

**15.b. If yes, how often do you think AF screening should be done?**

1= Once a year during an annual visit

2= At every visit as part of routine checking of vital signs

3= Other: ______________________________________________

1. **Do you think that patients at increased risk for AF (e.g., older patients) should be screened outside of office visits for persistent or paroxysmal AF, for example, by inviting patients to use a one-time 2-week patch monitor (e.g. Zio patch)?**

0= No

1= Yes

8= Unsure

1. **Do you think that patients at increased risk for AF should be screened outside of office visits for persistent or paroxysmal AF using personal consumer devices such as the Apple Watch, FitBit, or AliveCor Kardia mobile?**

0= No

1= Yes

8= Unsure

**17.a. If yes, should patients be able to send you results from such devices through a patient portal such as Patient Gateway or Epic MyChart?**

0= No

1= Yes

8= Unsure

1. **Have you ever recommended that a patient use an Apple Watch or AliveCor Kardia mobile device at home to determine if AF is present?**

0= No

1= Yes

1. **Consider a patient with a CHA_2_DS_2_-VASc score of 3 and paroxysmal AF. What is the minimum duration of a single AF episode that would lead you to recommend oral anticoagulation (OAC) for this patient? We acknowledge that decisions to prescribe OAC may be multifactorial.**

0= I don't prescribe OAC for paroxysmal AF

1= At least 30 seconds of AF

2= At least 5 minutes of AF

3= At least 3 hours of AF

4= At least 24 hours of AF

8= Unsure

1. **Please provide any additional feedback you have about the VITAL-AF study.**
